# The Use of Rituximab in Glomerulonephritis: What Is the Evidence?

**DOI:** 10.3390/biomedicines13092157

**Published:** 2025-09-04

**Authors:** Wenjuan Zhu, Haiyan He, Pearl Pai

**Affiliations:** 1 Department of Nephrology, University of Hong Kong-Shenzhen Hospital, Shenzhen 518000, China; zhuwj@hku-szh.org (W.Z.); hehy@hku-szh.org (H.H.); 2 Department of Medicine, University of Hong Kong LKS Faculty of Medicine, Pokfulam, Hong Kong

**Keywords:** rituximab, glomerular disease, nephrology

## Abstract

Rituximab has been increasingly used as a choice of treatment in various forms of glomerulonephritis. Recent evidence and the KDIGO guideline establish Rituximab as the first-line treatment for Primary Membranous Nephropathy and ANCA-associated vasculitis. It is also recommended in cases of steroid-dependent or frequently relapsing Minimal Change Disease and Focal Segmental Glomerulosclerosis, as well as in refractory Lupus Nephritis. In this review, we set out to analyze the evidence behind its use and some of its shortfalls, including gaps in understanding the dosing and redosing schedule, as well as long-term safety, which advocate for further research. Finally, we also provide some practical tips on patient use.

## 1. Introduction

Glomerulonephritis (GN) is an immune-mediated disorder characterized by inflammation of the glomeruli. This condition is primarily classified based on the histopathological patterns of injury [1]. Glomerular inflammation disrupts the renal filtration barrier, which leads to active urine sediments and/or renal injury [2]. Historically, the management of GN was limited to broad-spectrum immunosuppressants, including glucocorticoids and cytotoxic agents [3,4,5,6]. However, targeted biologic therapies have been developed as a result of recent advances in the understanding of its underlying immune mechanism. A key development in the field is the discovery of the pivotal role of B-lymphocytes in the development of numerous forms of autoimmune-mediated kidney disease.

### 1.1. The Pathogenic Role of B-Lymphocytes in Glomerular Injury

B-lymphocytes contribute to glomerular injury through several mechanisms. They differentiate into plasma cells that produce pathogenic autoantibodies. The immune complexes thus formed are deposited in the glomeruli and cause subsequent inflammation [7]. In addition, B-lymphocytes function as antigen-presenting cells (APCs). They are able to present self-antigens to autoreactive T-lymphocytes and enhance the autoimmune response [8].

The role of B-cell-derived cytokines is complex. Activated B cells may produce proinflammatory cytokines that can directly damage glomerular cells, including podocytes [9]. Conversely, certain B-cell subsets can secrete regulatory cytokines that may modulate or inhibit disease progression. This evidence suggests a dual role for B-lymphocytes in both promoting and regulating glomerular injury [7].

### 1.2. Rituximab’s Mechanisms of Action

Rituximab is a chimeric mouse–human IgG1 monoclonal antibody that targets the CD20 antigen. The CD20 antigen is expressed on B cells throughout their development but is lost once they become plasma cells [10]. Upon binding to CD20, rituximab mediates B-cell depletion through different pathways, including complement-dependent cytotoxicity (CDC), antibody-dependent cellular cytotoxicity (ADCC), and the induction of apoptosis [11]. Following a single course of rituximab, B cells remain depleted from the peripheral blood for about six to twelve months [10].

Beyond its role in B-cell depletion, rituximab may have direct, non-immunologic effects on podocytes. Podocytes are crucial for maintaining the integrity of the glomerular filtration barrier. Hence, podocyte injury is a primary cause of proteinuria. Rituximab stabilizes the podocyte cytoskeleton by preserving sphingomyelin phosphodiesterase acid-like 3b (SMPDL3b) and protecting the cytoskeleton [12]. This direct cytoprotective effect is particularly relevant in proteinuric kidney diseases.

## 2. The Use of Rituximab in Different Types of Glomerulonephritis

### 2.1. Primary Membranous Nephropathy

Primary Membranous Nephropathy (PMN) is a major cause of nephrotic syndrome in adults, responsible for about 20% of diagnoses [13]. The progression of PMN varies among patients. About 30–40% of patients’ conditions may resolve spontaneously, but approximately a third may progress to kidney failure [14]. The discovery of autoantibodies targeting the M-type phospholipase A2 receptor (PLA2R) established PMN as an autoimmune disease [15]. Circulating anti-PLA2R antibodies are now being used as a highly specific biomarker in the diagnosis and monitoring of disease activity. A decline in anti-PLA2R antibody levels reliably predicts subsequent clinical remission and offers a solid basis for B-cell-targeted therapy.

The standard of care for PMN comprised supportive therapy and the use of renin–angiotensin system (RAS) inhibition. Before the widespread use of rituximab, the 2011 Kidney Disease: Improving Global Outcomes (KDIGO) guideline recommended using cyclophosphamide or a calcineurin inhibitor in patients at high risk of disease progression [16].

#### The Landmark Randomized Controlled Trials of Rituximab

The GEMRITUX trial [2017] evaluated the efficacy of adding rituximab to non-immunosuppressive antiproteinuric treatment in patients with PMN and persistent nephrotic syndrome. Rituximab was administered using a standard dose (375 mg/m^2^) in two separate infusions [17]. At the primary endpoint of 6 months, there was no statistically significant difference in remission rates between the rituximab and control groups. However, a post hoc analysis with an extended period of follow-up (median, 17 months) revealed a significantly better remission rate in the group treated with rituximab (64.9% vs. 34.2%; *p* < 0.01).

The MENTOR trial provided further evidence for the delayed but durable efficacy of rituximab [18]. In this study, participants were assigned to receive either rituximab or a standard cyclosporine regimen. The rituximab protocol involved two 1 g infusions given 14 days apart. A repeat course was offered at 6 months in those with a partial response. At 12 months, rituximab was shown to be non-inferior compared with cyclosporine in inducing remission (60% vs. 52%). However, at 24 months, one year after the cessation of therapy, rituximab was superior to cyclosporine in maintaining remission (60% vs. 20%).

The STARMAN trial studied the sequential effect of 6-month tacrolimus (and tapered) followed by a stat dose of rituximab at 6 months versus 6 months of cyclical cyclophosphamide and corticosteroid therapy in PMN patients at high risk of progression [19]. The primary trial outcome was remission at 24 months. However, in this study, the cyclophosphamide corticosteroid regimen was superior in achieving remission (83.7% vs. 58.1%).

### 2.2. ANCA-Associated Vasculitis

ANCA-associated vasculitis (AAV) is a systemic autoimmune disease with characteristics of necrotizing inflammation affecting the small blood vessels. The two main clinical phenotypes are granulomatosis with polyangiitis (GPA) and microscopic polyangiitis (MPA) [20]. The disease is marked by the presence of anti-neutrophil cytoplasmic antibodies (ANCAs), which target either proteinase 3 (PR3-ANCA) or myeloperoxidase (MPO-ANCA) [21]. For decades, cyclophosphamide and glucocorticoids have been used as standard therapy for inducing remission.

#### Evidence from Randomized Controlled Trials

In AAV, rituximab has established itself as standard care for both disease induction and maintenance. RAVE is a pivotal trial that compared the efficacy of rituximab and cyclophosphamide in inducing remission in ANCA vasculitis. Patients received either i.v. rituximab (375 mg/m^2^) weekly for 4 weeks or daily oral cyclophosphamide (2 mg/kg). Both groups were also given glucocorticoids that were tapered off. The primary endpoint was disease remission without prednisone at the end of 6 months. Rituximab was non-inferior to cyclophosphamide for the primary endpoint (64% vs. 53%; *p* < 0.001 for non-inferiority). In patients with a previous relapse, rituximab demonstrated superiority over cyclophosphamide. (67% vs. 42%; *p* = 0.01) [22].

More recently, the combined use of rituximab and avacopan (a C5a receptor inhibitor) has demonstrated significant glucocorticoid sparing in the induction management of AAV [23].

As to maintenance, the effectiveness of rituximab for maintaining remission in AAV is particularly compelling. In the MAINRITSAN trial [24], AAV patients who had achieved remission with cyclophosphamide and glucocorticoids were assigned at random to a different maintenance therapy. Participants received either i.v. rituximab (500 mg on days 0 and 14, then infusions at 6, 12, and 18 months) or daily azathioprine until month 22. At 28 months, rituximab was shown to be significantly more effective than azathioprine in preventing a major relapse (5% vs. 29%; *p* = 0.002). (5% vs. 29%; *p* = 0.002).

The RITAZAREM trial further confirmed the superiority of rituximab in patients with relapsing AAV [25]. In this study, all patients received induction therapy with rituximab and glucocorticoids. Patients who achieved remission were then randomized to maintenance therapy with either scheduled rituximab infusions (1 g every 4 months) or azathioprine and followed up for at least 3 years. The trial was prematurely terminated after rituximab showed significant superiority over azathioprine in preventing relapse (HR 0.41; 95% CI 0.27 to 0.61; *p* < 0.001). At 24 months, relapse occurred in 13% of patients receiving rituximab versus 38% of the azathioprine group. These two trials have established rituximab as a highly effective agent for maintaining remission in AAV.

### 2.3. Podocytopathies: Minimal Change Disease and Primary Focal Segmental Glomerulosclerosis

Minimal change disease (MCD) and primary Focal Segmental Glomerulosclerosis (FSGS) are podocytopathies and leading causes of adult nephrotic syndrome. Historically, these conditions were thought to be mediated by T-cell dysfunction and a circulating “permeability factor” that injures podocytes, leading to the effacement of foot processes and massive proteinuria [26]. However, more recent evidence has implicated B-cell autoimmunity in their pathogenesis.

The discovery of anti-nephrin autoantibodies in a subset of patients with MCD provides a direct link to B-cell-mediated disease [27]. Although anti-nephrin antibodies are rare in primary FSGS [28], the presence of B-cell infiltrates in renal biopsies and the identification of other potential autoantibodies suggest that B cells may contribute to the pathogenesis in a subset of primary FSGS patients [29,30]. It is crucial to distinguish primary immune-mediated FSGS from secondary and genetic forms, as rituximab is unlikely to be effective in the non-immune types [31].

#### Evidence from Observational Studies and Meta-Analyses

The evidence for rituximab use in adult MCD and primary FSGS is based on observational studies and meta-analyses, since proper randomized controlled studies are lacking. These studies consistently showed that rituximab effectively reduced steroid use in cases of frequently relapse (FR) or steroid-dependence (SD). However, efficacy differs significantly according to the renal histology. In a meta-analysis [32] encompassing 21 studies and 382 adults with MCD/FSGS, 91.6% of the MCD patients achieved complete remission after 12 to 43 months, compared with only 43% of patients with primary FSGS. Another meta-analysis of 16 studies containing 221 patients with FR/SD disease found similar results, with complete remission rates of 74.7% in MCD versus 42.9% in FSGS [33]. Long-term observational data also showed that rituximab could reduce relapse rates and prolonged relapse-free survival in patients with podocytopathies [34]. These studies confirm the efficacy of rituximab in maintaining remission and reducing steroid burden in difficult-to-treat MCD, but its efficacy in FSGS is modest.

### 2.4. Lupus Nephritis

Lupus Nephritis (LN) is a severe organ manifestation of systemic lupus erythematosus (SLE) [35] and is a major cause of morbidity and mortality [36,37,38]. LN often occurs within five years of diagnosis of SLE or upon presentation [39]. Despite improvement in treatment, some patients with LN still experience disease progression and end up with kidney failure [40].

The pathogenesis of LN is a prototypical example of immune complex-mediated glomerulonephritis. The loss of self-tolerance leads to B-cell activation and the production of autoantibodies, resulting in immune complex formations that deposit in the glomeruli [41]. The resulting inflammation and cellular proliferation lead to different renal pathologies. The International Society of Nephrology/Renal Pathology Society (ISN/RPS) has categorized LN into six classes. The proliferative forms of LN (Class III and IV) are characterized by their aggressive nature and carry the highest risk of progression to end-stage kidney disease (ESKD) [42]. In view of the pivotal role of B-cell-derived autoantibodies in the pathogenesis of LN, B-cell depletion with rituximab is a highly rational therapeutic strategy.

#### 2.4.1. Evidence from Randomized Controlled Trials

The LUNAR trial was a randomized controlled trial assessing the use of rituximab in patients with proliferative LN (Class III or IV) [43]. Participants were randomly assigned to receive either rituximab or placebo, in the background of mycophenolate mofetil (MMF) and corticosteroid therapy. The main endpoint of the study was the overall renal response rates at 52 weeks. The study yielded negative results with no significant difference in 52-week renal response rates between rituximab and placebo (56.9% vs. 45.8%). Despite the failure of the LUNAR study to meet its primary clinical endpoint in LN, rituximab has demonstrated clear biological efficacy, including significant improvement in serological markers (reduced anti-dsDNA and increased levels of complement C3/C4). The contradictory results are perplexing. It has been hypothesized that the high efficacy of the background immunosuppressive regimen might have obscured the potential benefit of rituximab [44]. Moreover, the primary endpoint selected may have lacked sensitivity in capturing all clinically meaningful improvements.

#### 2.4.2. Evidence in Refractory LN

Unlike the LUNAR trial, observational studies and meta-analyses supported the use of rituximab in patients with LN that failed to respond to standard therapies. A meta-analysis of 24 studies and 940 patients demonstrated that rituximab significantly improved both total remission (TR: complete remission plus partial remission) (OR = 2.02, 95% CI: 1.23–3.32) and complete remission (OR = 1.98, 95% CI: 0.90–4.39) compared to the control groups (MMF or cyclophosphamide) [45].

A systematic review of 300 patients with refractory LN provided further evidence regarding the efficacy of rituximab [46]. The study reported a high rate of partial or complete remission across different histological classes: 87% for Class III, 76% for Class IV, and 67% for Class V. The corresponding rates of complete remission were 60%, 45%, and 40%, respectively. These analyses supported the role of rituximab in LN patients who failed to respond to standard immunosuppressive therapies.

## 3. Using Rituximab in Practice: Some Tips and Special Attention

In clinical practice, rituximab is usually given according to two regimens. Standard rituximab induction regimens involve either four weekly infusions of 375 mg/m^2^ or two infusions of 1 g given at 2-week intervals. The subsequent dose is usually given at regular intervals (e.g., after 6 or 12 months), depending on the center’s experience and the patient’s response. Some centers monitor the total peripheral CD19+ B-cell counts to guide redosing, but this is neither sensitive nor specific.

### 3.1. Safety and Risk

Although rituximab has a more favorable safety profile than cyclophosphamide, it is a potent immunosuppressant with specific risks [47]. Acute infusion reactions are common adverse events, occurring in a significant proportion of patients during the first infusion. Symptoms can include fever, chills, rigors, flushing, rash, bronchospasm, and hypotension, but are rarely life-threatening [48].

Serious infection may occur following rituximab as a result of prolonged B-cell depletion, hypogammaglobulinemia, and late-onset neutropenia, which lead to greater susceptibility to infections. Serious opportunistic infections may occur, including pneumocystis jiroveci pneumonia (PJP), progressive multifocal leukoencephalopathy, and flare-ups of hepatitis B (HBV) and tuberculosis [49]. Prophylactic strategies and careful monitoring are essential. This involves pre-therapy screening for viral hepatitis and tuberculosis. Monitoring immunoglobulin levels is crucial before and after rituximab infusions to identify at-risk patients and guide treatment decisions [50,51,52].

### 3.2. Management of Non-Responders

Approximately 20–40% of patients may fail to respond to rituximab or experience a relapse. The mechanisms of resistance may be due to pharmacokinetic (PK) or pharmacodynamic (PD) variations.

PK failure occurs when the drug exposure is insufficient to achieve effective B-cell depletion [53]. PK failure is most often seen in conditions that increase rituximab clearance, such as loss of the drug in the urine in patients with heavy proteinuria [54]. PK failure is also seen in patients who develop anti-drug antibodies, leading to faster B-cell reconstitution and secondary treatment failure upon re-exposure [55].

The occurrence of PD failure is contingent upon the presence of long-lived, CD20-negative plasma cells that evade targeting by rituximab [56] or if the disease is not primarily B-cell-mediated, as may be the case in certain forms of FSGS.

In order to manage the non-responders systemically, it is essential to confirm if adequate B-cell depletion has been achieved by measuring peripheral CD19+ counts. If depletion is incomplete and PK failure is suspected, measuring rituximab drug levels can be informative. In cases of secondary failure, testing for anti-rituximab antibodies may be warranted. Therapeutic pivots for non-responders include dose intensification, more frequent dosing, or a switch to an alternate agent. For patients with suspected immunogenicity, switching to a humanized (e.g., obinutuzumab) or fully human (e.g., ofatumumab) anti-CD20 antibody may address resistance in patients with suspected immunogenicity [56].

## 4. Discussion

Based on current evidence, the KDGIO guideline has made the following recommendations. In the 2021 KDIGO guideline for PMN, it is recommended that rituximab be used as first-line therapy in patients with a moderate or high risk of progression to ESKD. For moderate-risk patients, the guideline recommends combining rituximab with glucocorticoids. For high-risk patients, the recommended treatment options include rituximab monotherapy or combining rituximab with a calcineurin inhibitor (CNI) [57]. The combination therapy with CNI brings together two distinct drug profiles. The CNIs should bring about rapid control of proteinuria by stabilizing the podocyte cytoskeleton while waiting for the rituximab to produce a more long-lasting remission [57].

Also in the KDIGO guideline for PMN, it specifically refers to the monitoring of PLA2R antibody after 6 months. This is because, following a standard rituximab regimen, there is usually a sustained depletion of circulating CD20+ B cells for 6–12 months [58] together with a disruption of PLA2R antibody production. Since the immunological response reliably precedes the clinical response, monitoring the PLA2R antibody at 6 months can be used to guide further treatment. Similarly, it has been shown in the Mentor trial that the PLA2R antibody levels at 6 months were a strong predictor of proteinuria remission at 12 months [59].

The 2024 KDIGO guideline for AAV has endorsed the use of rituximab as a primary therapy to induce and maintain remission [60]. In new-onset, organ-threatening AAV, the recommendation is to use either rituximab or cyclophosphamide, in combination with glucocorticoids, as a first-line agent. Rituximab is the preferred choice for patients with relapsing disease, who are PR3-ANCA-positive, or when preserving fertility is important. Rituximab is also preferable as a maintenance therapy over azathioprine. This indication is particularly strong for patients at high risk of relapse, especially those with PR3-ANCA. The use of rituximab with cyclophosphamide has been reserved for controlling the most severe forms of AAV and rapidly progressive glomerulonephritis.

The 2021 KDIGO guideline recommends the use of high-dose glucocorticoids as a first-line therapy for the initial treatment of both MCD and primary FSGS [57]. But in cases with FR/SD disease, rituximab is recommended as an alternate therapy to calcineurin inhibitors or MMF, particularly in MCD. The determination of treatment should be personalized based on prior therapies and patient-specific factors. The potential use of anti-nephrin antibodies as a specific biomarker in primary podocytopathies needs further research and evaluation.

Taking into consideration the available evidence and the negative result of the LUNAR trial, the 2024 KDIGO guideline on LN [61] has not recommended using B-cell depletion therapy or rituximab as part of the initial therapy. However, in cases of refractory proliferative LN, the guideline refers to a change in immunosuppressive therapy, including the use of rituximab. The KDIGO guideline also suggests combining rituximab and glucocorticoids with other immunosuppressants (MMF or cyclophosphamide) in the treatment of severe and refractory types of LN where other therapies have failed [61]. The KDIGO guideline recommendations for rituximab use in glomerulonephritis are summarized in Table 1.

At this time, there is no evidence about combining rituximab with other monoclonal antibodies in the treatment of primary glomerular disease, but the use of rituximab and belimumab has been studied in patients with SLE [62]. However, the phase 3 BLISS-BELIEVE trial did not meet its primary endpoint of superior disease control even though patients receiving the combination therapy showed a greater reduction in anti-dsDNA antibodies.

The guide for redosing rituximab is still unclear. Rituximab specifically targets CD20-expressing B cells and depletes B cells profoundly in the peripheral blood. However, little is known about the B-cell depletion in the deeper tissues. Biomarkers, including CD19+ B cells or CD27+ B cells in the peripheral blood, have been used to guide rituximab redosing in different autoimmune diseases such as rheumatoid arthritis [63], with varying success. Memory B cells are integral components of immunological memory and provide the reservoir of autoimmunity. There is some evidence that memory B cells may be a useful biomarker to guide retreatment [63]. Future studies should focus on memory B-cell phenotypes as a biomarker.

Even though rituximab has become a key treatment for many types of primary GN, there are limitations, including immunogenicity and incomplete B-cell depletion. These concerns have driven the development of next-generation anti-CD20 antibodies that are more potent but less immunogenic. Obinutuzumab is a glycoengineered, humanized Type II, anti-CD20 monoclonal antibody [64]. Unlike rituximab, which relies heavily on complement-dependent cytotoxicity (CDC), obinutuzumab induces more potent direct cell death and antibody-dependent cellular cytotoxicity (ADCC) compared with rituximab [65]. Hence, obinutuzumab stands as a promising alternative therapy for rituximab-refractory MN and MCD [66].

Ofatumumab’s fully human structure minimizes the risk of immunogenicity, making it an attractive option for patients who have developed intolerance (e.g., infusion reactions) or immunogenic resistance to rituximab [67]. Meanwhile, because of a slower off-rate, ofatumumab may lead to more effective CDC [68]. Case series have reported its successful use in rituximab-refractory AAV, MN, and LN [69,70,71].

## 5. Conclusions

The use of rituximab in the treatment of certain types of primary glomerulonephritis has changed the scene of its management in recent years. Several landmark studies have confirmed its place as a first-line therapy in PMN and ANCA-associated vasculitis. Its use in steroid-dependent or frequently relapsing MCD or FSGS and refractory LN is also gaining attention. However, gaps in understanding the mechanism of rituximab remain, and more research is needed to enable an effective dosing regimen and personalized therapy.

## 6. Future Directions

Despite its widespread use in various forms of glomerulonephritis, the optimal dosing and redosing schedule of rituximab is unclear. In this regard, prospective trials are necessary to establish the optimal dosage, treatment duration, and redosing strategy (fixed vs. biomarker-guided) for rituximab and other biologics. In the same light, more research is needed to search for biomarkers that can accurately predict treatment response or relapse, guide redosing, and enable personalized therapy. We believe establishing a long-term safety registry for rituximab users over an extended period will help clinicians and researchers to understand its safety in long-term use. Finally, future trials comparing the efficacy and safety of rituximab versus next-generation agents will provide information on how we can best use these drugs in practice.

## Figures and Tables

**Table 1:** KDIGO guideline recommendations for rituximab use in glomerulonephritis.

Disease	KDIGO Guideline Recommendation	Key Evidence	Key Findings
Primary Membranous Nephropathy (PMN)	First-line therapy for patients at moderate or high risk of progression to ESKD [57].	GEMRITUX Trial [17]	Extended follow-up showed a significantly higher remission rate in the rituximab group (64.9% vs. 34.2%).
		MENTOR Trial [18]	At 24 months, rituximab was superior to cyclosporine in maintaining remission (60% vs. 20%).
ANCA-Associated Vasculitis (AAV)	Recommended as a key agent for both induction and maintenance of remission. Preferred for relapsing disease and as maintenance therapy over azathioprine [60].	RAVE Trial [22]	Non-inferior to cyclophosphamide for induction; superior in patients with relapsing disease (67% vs. 42%).
		MAINRITSAN Trial [24]	More effective than azathioprine in preventing major relapse at 28 months (5% vs. 29%).
		RITAZAREM Trial [25]	Overwhelmingly superior to azathioprine in preventing relapse in patients with relapsing AAV (HR 0.41).
Podocytopathies (MCD and FSGS)	Recommended as an alternative therapy for frequently relapsing or steroid-dependent (FR/SD) disease, particularly for MCD [57].	Meta-analysis [32]	Efficacy differs significantly by histology. Complete remission achieved in 91.6% of MCD patients vs. 43% of primary FSGS patients.
Lupus Nephritis (LN)	Not recommended as part of initial therapy. May be considered in cases of refractory proliferative LN (2024 KDIGO guideline) [61].	LUNAR Trial [43]	Failed to meet its primary endpoint; no significant difference in overall renal response rate vs. placebo (56.9% vs. 45.8%).
		Meta-analysis (Refractory LN) [45]	In refractory LN, rituximab significantly increased total remission (OR = 2.02) and complete remission (OR = 1.98) vs. control groups.

Legend: AAV, ANCA-associated vasculitis; ESKD, end-stage kidney disease; FR/SD, frequently relapsing or steroid-dependent; FSGS, Focal Segmental Glomerulosclerosis; HR, Hazard Ratio; KDIGO, Kidney Disease: Improving Global Outcomes; LN, Lupus Nephritis; MCD, Minimal Change Disease; OR, Odds Ratio; PMN, Primary Membranous Nephropathy.

## Data Availability

The original contributions presented in this study are included in the article. Further inquiries can be directed to the corresponding author.
